# Draft Genome Sequence of *Pseudenhygromyxa* sp. Strain WMMC2535, a Marine Ascidian-Associated Bacterium

**DOI:** 10.1128/MRA.00657-20

**Published:** 2020-08-20

**Authors:** Shaurya Chanana, Doug R. Braun, Scott R. Rajski, Tim S. Bugni

**Affiliations:** a Pharmaceutical Sciences Division, School of Pharmacy, University of Wisconsin, Madison, Wisconsin, USA; University of Delaware

## Abstract

*Pseudenhygromyxa* WMMC2535, a representative of the myxobacteria (family *Nannocystaceae*), was isolated from a ragged sea hare in the Florida Keys, and its genome was sequenced using PacBio technology. The WMMC2535 genome sequence is the first of this genus and validates the notion that myxobacteria represent outstanding sources of structurally diverse natural products.

## ANNOUNCEMENT

Myxobacteria, especially those of a marine origin, display the potential to serve as prolific producers of natural products (NPs) with novel scaffolds, although their amenability to laboratory cultivation is a clear limitation (1–11). The draft genome sequence of strain WMMC2535, from the genus *Pseudenhygromyxa*, is only the 7th example of a marine myxobacterium genome (8). An NCBI BLAST search revealed that the 16S rRNA sequence of WMMC2535 is 97.13% identical to that of Pseudenhygromyxa salsuginis, the only previously described member of the genus. Notably, *P*. *salsuginis* has not been sequenced, thus making WMMC2535 the first member of this rare genus to have its genome sequenced. Unlike *P. salsuginis*, the WMMC2535 isolate is halophilic rather than halotolerant (12). Further analysis of the ∼9.5-Mb genome using antiSMASH 5.0 (13) provides insight into this strain’s immense biosynthetic potential.

WMMC2535 was isolated in 2016 from the digestive tract of a ragged sea hare (Bursatella leachii) collected in the Florida Keys (24 39′29.4ʺ, –81 25′15.1ʺ) as part of an ongoing drug discovery campaign. Isolation was performed using the baiting technique described by Iizuka et al. (14), and a pure culture was maintained on medium containing 1% casein and 1.5% agar with 50% artificial seawater (ASW) (15). The 16S rRNA gene was amplified using the primers 8-27F (5′-GAGTTTGATCCTGGCTCAG-3′) and 1492R (5′-GGTTACCTTGTTACGACTT-3′). Cells were scraped off solid medium and diluted into 20 μl of Milli-Q water. A 2-μl aliquot of this dilution was added directly to the PCR mix. 16S rRNA sequencing readily revealed WMMC2535 to be a member of the genus *Pseudenhygromyxa*, belonging to the family *Nannocystaceae*.

Cells of *Pseudenhygromyxa* sp. WMMC2535 were scraped off a plate of medium containing 1% casein, 0.5 mg cyanocobalamine liters^−1^, 1.5% agar, and 50% artificial seawater (ASW) and inoculated into liquid medium of the same composition. After 1 week of shaking at 28°C and 200 rpm, cells were centrifuged for DNA extraction. DNA was isolated using a standard phenol-chloroform extraction and then purified using the DNeasy PowerClean cleanup kit (Qiagen). The DNA was size sorted using BluePippin (Sage Science) with an insert target size of 15 to 20 kb. The library was prepared with the SMRTBell template prep kit v1.0 (PacBio) and sequenced on V3 chemistry. Next-generation sequencing was performed at 2,055× coverage using a PacBio Sequel platform (University of Wisconsin, Madison [UW-Madison], Biotechnology Center). Two Sequel single-molecule real-time (SMRT) cells produced 18,499,171,043 bp in 1,370,320 reads. PacBio data were corrected, trimmed, and assembled into 42 contigs with Canu v1.8 (16) using the parameter “genomeSize=9m.” An NCBI BLAST search revealed two contigs as mitochondrial DNA and one as a PacBio adapter sequence, which were removed. QUAST v5.0.2 (17) was used to assess the assembly quality parameters of the remaining contigs, finding that the WMMC2535 genome contains a total of 10,191,795 bp with a GC content of 69.63%, *N*_50_ and *N*_75_ values of 9,528,923 bp, *L*_50_ and *L*_75_ values of 1, and a maximum contig size of 9,528,923 bp.

This organism’s secondary metabolic potential was assessed using antiSMASH and was found to contain up to ∼30 different biosynthetic gene clusters (BGCs), 7 of which were identified on the basis of percent identity to established BGCs. Two type I polyketides (T1-PKS), one siderophore (desferrioxamine), two terpenes, one aryl polyene (APE V_f_), and one hybrid T1-PKS-heterocyst glycolipid synthase-like PKS (encoding eicosapentaenoic acid) BGC were identified. Hence, the genome analysis of WMMC2535 supports the notion that myxobacteria represent excellent repositories for natural product biosynthetic machineries and capabilities.

### Data availability.

The genome sequence of *Pseudenhygromyxa* sp. strain WMMC2535 has been deposited in GenBank under accession number CP049288 (BioSample number SAMN14168466 and BioProject number PRJNA608260). The raw reads can be accessed under SRA number SRP259651.
